# Cycle-specific female preferences for visual and non-visual cues in the horse (*Equus caballus*)

**DOI:** 10.1371/journal.pone.0191845

**Published:** 2018-02-21

**Authors:** Dominik Burger, Charles Meuwly, Selina Thomas, Harald Sieme, Michael Oberthür, Claus Wedekind, Sabine Meinecke-Tillmann

**Affiliations:** 1 Swiss Institute of Equine Medicine ISME, University of Berne, and Agroscope, Avenches, Switzerland; 2 Unit for Reproductive Medicine–Clinic for Horses, University of Veterinary Medicine Hannover, Hanover, Germany; 3 Institute for Reproductive Biology, University of Veterinary Medicine Hannover, Hanover, Germany; 4 Department of Ecology and Evolution, Biophore, University of Lausanne, Lausanne, Switzerland; University of Missouri Columbia, UNITED STATES

## Abstract

Although female preferences are well studied in many mammals, the possible effects of the oestrous cycle are not yet sufficiently understood. Here we investigate female preferences for visual and non-visual male traits relative to the periodically cycling of sexual proceptivity (oestrus) and inactivity (dioestrus), respectively, in the polygynous horse (*Equus caballus*). We individually exposed mares to stallions in four experimental situations: (i) mares in oestrus and visual contact to stallions allowed, (ii) mares in oestrus, with blinds (wooden partitions preventing visual contact but allowing for acoustic and olfactory communication), (iii) mares in dioestrus, no blinds, and (iv) mares in dioestrus, with blinds. Contact times of the mares with each stallion, defined as the cumulative amount of time a mare was in the vicinity of an individual stallion and actively searching contact, were used to rank stallions according to each mare’s preferences. We found that preferences based on visual traits differed significantly from preferences based on non-visual traits in dioestrous mares. The mares then showed a preference for older and larger males, but only if visual cues were available. In contrast, oestrous mares showed consistent preferences with or without blinds, i.e. their preferences were mainly based on non-visual traits and could not be predicted by male age or size. Stallions who were generally preferred displayed a high libido that may have positively influenced female interest or may have been a consequence of it. We conclude that the oestrous cycle has a significant influence on female preferences for visual and non-visual male traits in the horse.

## Introduction

Female preferences for male characteristics are expected to be driven by different selective forces and may hence often be context dependent [1]. Among the selective forces that usually shape female preferences are direct benefits (e.g. parental care, protection, resource acquisition, parasite avoidance) and indirect benefits (e.g. good or compatible genes passed on to the common offspring) [2–5]). Females therefore often base their choice on various traits that may signal different male characteristics, for example, olfactory signals (mouse, *Mus musculus* [6]), vocal signals (red deer, *Cervus elaphus* [7]), behavioural and visual displays (the chest stain in Verreaux’s sifaka, *Propithecus verreauxi* [8]), including the size and complexity of secondary sexual traits (the antlers in male red deer, *Cervus elaphus* [9], or the horn size of wild bighorn rams, *Ovis canadensis* [10]). The context dependency of female preferences may then be given by the relative importance of the male characteristics that are displayed.

There is a great diversity of well-studied exogenous factors that affect female preferences in mammals. Examples include territory characteristics occupied by the respective male (Uganda kob, *Kobus kob thomasi* [11,12]), protection from predation risk or harassment (fallow deer, *Dama dama* [13]), and male infanticide [14] (the equines *Equus caballus* [15,16] and *E*. *ferus przewalskii* [17]). The influence of endogenous factors like, for example, the oestrous cycle of female mammals, seems less intensely studied [18], despite the fact that the sexually receptive phase associated with high oestrogen production in the ovaries and ovulation (oestrus, “heat”) differs in so many aspects from the sexually non-active phase dominated by the formation of a progesterone secreting corpus luteum of the ovary after ovulation (luteal phase, dioestrus) [19]. So far, this differentiation has been taken into consideration in only a few non-human mammals including the domestic sheep, *Ovis aries* [20], domestic goats, *Capra hircus* [21], house mouse, *Mus musculus* [22], harvest mice, *Micromys minutus* [23], and African striped mice, *Rhabdomys pumilio* [24]. In humans, effects of menstrual cycle on mate preferences have been investigated in much detail (for reviews, see [25,26]). Women’s preferences seem to differ often between mid-cycle, i.e. during the time period of ovulation, and other times for facial masculinity [27], symmetry [28], body height [29], masculinity [28], or the scent of social dominance [30] and androstenone [31].

Here we investigate the possible link between female preferences and oestrous cycle in the horse (*Equus caballus*). Horses provide an excellent opportunity for studying female preferences because they are often used to handling, i.e. experimental work is comparatively easy. Under feral conditions, individuals either live in bands of breeding animals (“harems”) or of non-breeding stallions (“bachelors”) [32–34]. Harems consist of one to few harem stallions along with a herd of several breeding mares together with their offspring, “bachelor bands” fluctuate in size, often between two and 17 stallions. The horse is a seasonally poly-oestrous species, with ovulatory oestrous cycles typically occurring in spring and summer. The oestrous cycle is approximately 22 days in length, including an oestrous time period of 5–7 days each with an ovulation approximately 24 hours before the end of oestrus [35]. However, timing and frequency of oestrous cycles also depend on the presence or absence of a stallion [36]. During oestrus, mares show sexual interest for stallions with typical proceptivity towards them. In contrast, in the dioestrous luteal phase in-between oestrus, mares are observed to exhibit agonistic behaviour towards males or seem to be in general less interested in interacting with them [37].

In feral herds, the role of a harem stallion seems not to be the constant initiator of reproductive interactions, but primarily the provider of protection and herd cohesion. As reported in studies on other mammalian species such as elephant seals *Mirounga angustirostris*, greater horseshoe bats *Rhinolophus ferrumequinun*, house mice *Mus musculus*, red deer *Cervus elaphus*, fallow deer *Dama dama*, and grey house lemur *Microcebus murinus*, all reviewed in Clutton-Brock and McAuliffe [5], it is usually the mare that plays the decisive role in mate choice and initiates copulation [38]. The equine female is expected to be more selective than many other polygamous mammals because she provides much parental investment [38]. Indeed, individual mares in an oestrous state have been shown to exhibit distinct preferences for certain stallions [39,40]. It has also repeatedly observed that female dispersal leads to the creation of new bands (e.g. [41]). Dispersal probably aims for inbreeding avoidance (e.g. natal dispersal [42,43]) or may be a consequence of other types of mate preferences (e.g. after displacement of a stallion by another [44,45]), but may also depend on local animal density [41] and ecological conditions [46]. Temporary or permanent dispersal was observed in around 75% of the females per harem at frequencies of up to 18% per month [44] and 23% per year [41], respectively, beginning at the age of 1–2 years in young mares dispersing from their natal band, and subsequently but less between breeding bands, with peaks occurring in-between the breeding seasons (in dioestrus or anoestrus) and during the breeding periods in oestrus [43].

Recent analyses concluded that male and female preferences in the horse are influenced by the major histocompatibility complex (MHC; a set of genes that play a crucial role in immunity and in social signalling among vertebrates [47,48]): MHC-dissimilar mates are generally preferred [40,49,50,51]. However, when testing for MHC-dependent female preferences, Burger et al. [50] could find them only during dioestrus, not during oestrus, and it remained unclear what other type of signals influenced female preferences. Here we test whether there are cycle-specific female preferences for visual and non-visual male traits.

## Materials and methods

### Animals and infrastructures

Nineteen clinically healthy, normally cycling, non-pregnant mares without foals were investigated during one breeding season. They were of different breeds but with similar general phenotypic appearance (10 Warmblood, 4 Franches-Montagnes, 3 Standardbred, and 2 Thoroughbred horses) and breeding history (mares with one or more earlier foalings: *N* = 13, mean age ± SD = 9.7 ± 3.0 years, range 6–17 years; no previous foaling: *N* = 6, mean age ± SD = 6.5 ± 1.9 years, range 5–10 years). They were all owned by the Swiss National Stud in Avenches, Switzerland, and stabled there for more than 6 months in a structured group housing system with permanent access to a paddock and without any stallion contact. They received concentrated feed twice daily and hay and water *ad libitum*. None of the mares had exhibited any previous contact with the experimental stallions. All animals had been serotyped for parallel analyses on MHC-linked female preferences [50].

Each mare’s oestrous cycle was monitored daily when in oestrus and every three days during dioestrus, respectively, by transrectal ultrasonographic examination in the morning using an Aquila Pro VET (Esaote; Genova, Italy). Oestrous behaviour of the mares was monitored by teasing with a stallion that was not used in the experiment (see below). On the day when at least one follicle had reached a diameter ≥ 35 mm in the absence of a corpus luteum, a uterine oedema of stage 2 or higher was present [52] and oestrous behaviour was registered, the mares were injected intravenously with 1500 IU hCG (Chorulon 1500, Intervet; Boxmeer, Netherlands) in the evening to induce ovulation after approximately 36 hours, thereby enabling a standardized pre-ovulatory status of the mares in the experiment. The experiments took place on the following day after hCG-injection (“oestrus”) and five to twelve days after ovulation (“dioestrus”).

Seven unrelated stallions were used for these experiments. They were all from the Franches-Montagnes horse breed, with proven fertility, aged 5 to 19 years old (mean age ± SD = 12.7 ± 5.8 years), owned by the same owner and stabled at the stud for more than 2 years. They were kept in individual boxes bedded with straw, regularly and individually exercised, and had daily access to individual paddocks for approximately 1 hour a day. Care was taken to avoid any contact with mares apart from the controlled experimental situations. The stallions were fed 3 times daily with hay, oats, barley, corn, and pellets supplemented with minerals and had *ad libitum* access to water. All mares and stallions had been vaccinated and dewormed prior to the experiments.

**Fig 1:**
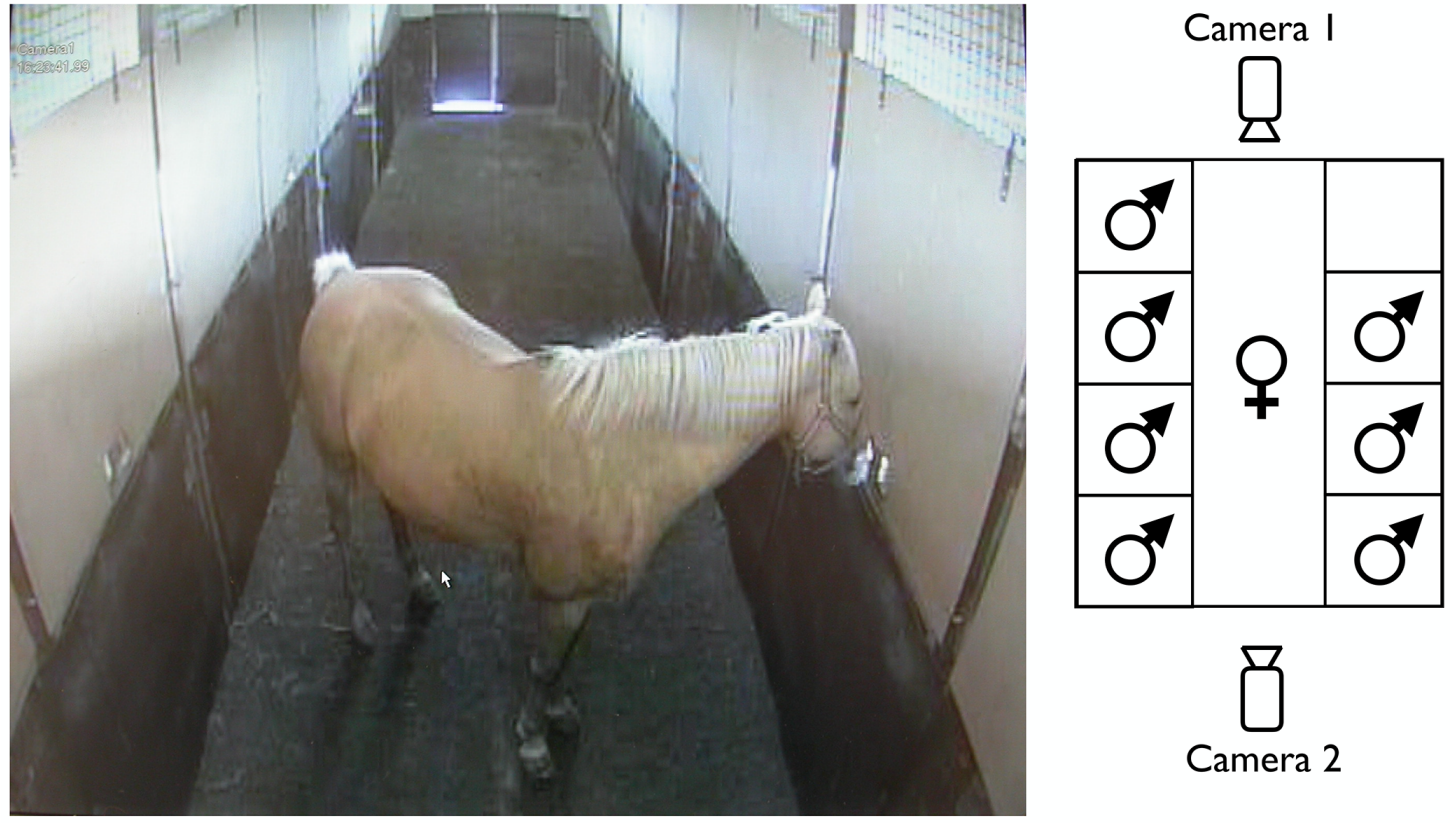
Experimental set-up. Exposure of stallions (in boxes behind blinds with small opening) to a mare (in corridor).

Experiments were performed using a specially designed stable (Fig 1) with 2 x 4 boxes of 12 m^2^ placed on either side of a 12.65 m long and 2.90 m wide corridor with non-slip rubber flooring. The walls of the boxes were constructed of solid wood up to a height of 1.40 m, with a metal grille above (total wall height: 3.65 m). This allowed visual, olfactory, and to a certain extent tactile contact between the animals. Their behaviour during the experiments was recorded with two cameras (Securiton, Securiton AG; Zollikofen, Switzerland, and Sony DCR VX 2000, Sony Electronics Inc.; Park Ridge, NJ, USA) positioned on each side of the corridor so that the mare’s behaviour could be observed outside the experimental room on an Intellex LT screen using the software Intellex 5.0 (American Dynamics; Boca Raton, FL, USA). Contact times of the mares with the stallions were monitored using a stopwatch (Certina DS, Certina S.A.; Le Locle, Switzerland).

### Experimental procedure

All tests were performed during the horses’ natural breeding season in spring and summer. We aimed at testing female preferences four times during two oestrous cycles, twice during oestrus and twice during dioestrus. The first two series of experiments took place during oestrus and dioestrus of the oestrous cycles between March 30^th^ and June 15^th^. The stallions’ box fronts were fully covered with wooden panels with the exception of a small opening of 21.5 x 15.0 cm towards the corridor, 1.50 m above the bottom of the box front, permitting olfactory and vocal contact but very limited visual contact (= non-visual tests with blinds; Fig 1). After having removed the blinds to allow full visual contact with the mares, the second two test series took place during later cycles (oestrous and dioestrous stages between June 10^th^ and August 7^th^).

On each experimental day and at least two hours before initiation of the tests, the stallions were randomly allocated to seven of the eight boxes (freshly bedded with straw each). Before each test series the mare was led into the corridor and walked clockwise along the seven stallion boxes starting at the first box to the left of the entrance stable door. While directing her along the box rows, she was halted in front of each stallion’s box with her head close to the small openings in the blinds, or to the stallion when the blinds had been removed. A nose to nose contact time of 15 seconds was allowed between the mare and each stallion.

Each test series consisted of several test rounds which were carried out as follows: the mare was positioned in the centre of the corridor and then released. The experimenter (C. Meuwly) then quickly left the stable, closed the stable door and used the remote video surveillance and a stopwatch to record the mare’s contact time towards each of the seven stallions (i.e. direct contact between the experimenter and the animals was avoided during the observational period). Contact time was defined as the cumulative amount of time (in seconds) the mare was in the vicinity (less than 10 cm) of an individual stallion’s box and actively searching nostril or body contact with the stallion. Agonistic behaviour, or behaviour that suggested that the mare was not interested in the stallions (for example looking out of the window of the stable door or standing indifferently in the corridor without actively searching contact) was interpreted and recorded as ‘disinterest’. Ten minutes after the mare had been released in the corridor, the mare was temporally placed into another stable without any contact to other horses, and the stallion with the highest cumulative contact time was removed from the stable. The test procedure was then immediately repeated until only two stallions remained in the last test round or until a mare showed no behaviour during a 10-minutes round that could be interpreted as interest in any of the stallions.

The stallions were scored on a scale from 1 to 7 with regard to the choice of each mare, with a preference score of 1 for the stallion that received the highest cumulative contact time in the first test round, and a score of 7 for the stallion that never reached the highest cumulative contact time and hence remained in the last test round when only 2 stallions were present in the experimental stable. Tied ranks were assigned to all remaining stallions if a female showed no behaviour that could be interpreted as interest in any of the stallions during a 10-minute test round.

Investigative and affiliative behaviours (stance, hind legs posturing, tail raising, clitoris winking, urinating, searching for stallion contact with nose or pelvic area, remaining calm, tolerance of stallion’s behaviour as sniffing and nibbling) and agonistic behaviours (rearing, kicking, tail switching, biting, threatening, position moving) toward each stallion were evaluated retrospectively using video analysis according to a specifically designed ethogram (adapted from Waring [53] and Crowell-Davis [37]). If two mares were tested on the same day, the corridor of the experimental stable was cleaned in between.

Phenotypic traits of each stallion were determined by registering body height (distance from the ground to the withers in cm), weight (kg), body condition score (1 = emaciated to 5 = obese) (modified from Carroll and Huntington [54] and Henneke et al. [55]), as well as the density of mane and tail hair (1 = sparse, 2 = medium, 3 = dense). Coat colour was not included in the analyses because all stallions were either chestnut or brown. On four occasions each, a minimum of 1 week apart, ejaculates of all stallions were collected on a dummy (without the presence of a mare) or, if not possible, in presence of or on an oestrous mare in order to assign a libido score (0 = no libido to 4 = high libido) [56].

Three weeks before the end of the study, a 15-year old stallion had to be euthanized due to an acute colic. As he seemed as healthy as the other stallions during the experiments until onset of the colic, all measurements until his death were included in the analyses. Three of the 19 mares were only available during the first two experimental series (test with blinds, mares in oestrus and in dioestrus).

### Statistics

Statistics were performed using JMP (Version 9, SAS Institute Inc., Cary, NC, 1989–2007) and the statistical software R version 2.14.1 (R Development Core Team 2010). Results are presented as means with the standard deviation (SD). Spearman rank order correlation coefficients (r_s_) were used to test for correlations, Wilcoxon signed rank tests for within-group comparisons, and Kruskal-Wallis test for between-group comparisons. All *P*-values were two-tailed and significant if < 0.05.

### Ethical note

Animal experimentation was performed following approval from the local Animal Ethics Committee (*Etat de Vaud*, *Service vétérinaire*, permit #2211).

## Results

During oestrus the contact time of mares in the first test round with the most preferred stallion each was on average 228 ± 128 s with blinds (range: 25–513 s) and 178 ± 47 s without blinds (range 123–265 s), during dioestrus 93 ± 110 s with blinds (range: 0–387 s) and 151 ± 109 s without blinds (range: 14–460 s). Differences to the next ranked stallion varied in oestrous mares from 8 to 504 s with blinds and 2 to 177 s without blinds, respectively. Mares took 12.5 ± 9.9 s (without blinds) and 36.2 ± 22.8 s (with blinds) during oestrus and 66.4 ± 48.6 s (without blinds) and 101.3 ± 86.4 s (with blinds) during dioestrus to instigate a first contact with a stallion. The order in which the males were presented to the mares had no effect on the latter’s preferences. More detailed results are shown in S1 Table and S2 Table.

**Fig 2:**
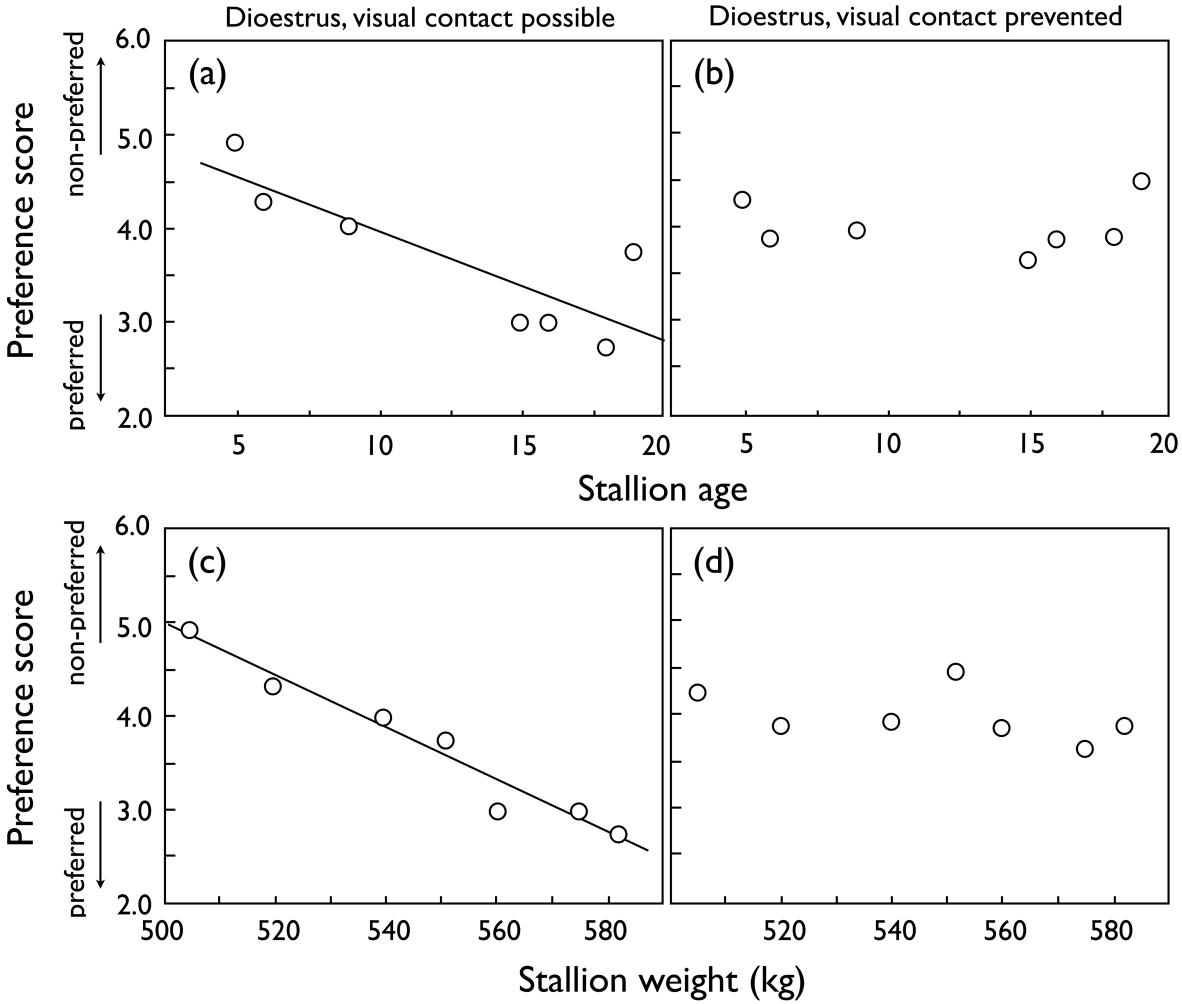
Preferences of dioestrous mares. Mean preference scores (1 = very attractive to 7 = not attractive) of dioestrous mares for stallions of different age when (a) visual contact was possible (r_s_ = -0.77, *P* = 0.04), and (b) visual contact was prevented (r_s_ = 0.13, *P* = 0.79). The corresponding correlations to stallion weight are given in panels (c) (r_s_ = -0.99, *P* < 0.001) and (d) (r_s_ = -0.41, *P* = 0.36), respectively. Regression lines are drawn to illustrate significant correlations.

The stallions’ age, body height and weight, body condition score, mane hair density score, and the libido score are listed in Table 1. Stallion body weight was positively correlated to body height (N = 7, r_s_ = 0.76, *P* = 0.05) and body condition score (N = 7, r_s_ = 0.85, *P* = 0.02). Female preferences for male size and age depended on the stage of their oestrous cycle. Dioestrous females showed a strong preference for the proximity of older males and heavier ones (Fig 2A and 2C). These preferences appeared to be based on visual traits because no correlation to male age or weight could be found when blinds prevented visual contact (Fig 2B and 2D). The density of the male’s mane and tail hair was never correlated to female preference (|r_s_| always < 0.29, *P* > 0.53).

**Table 1:** Age, body height, body weight, body condition score (range: 1–5), mane density score (1 = sparse, 2 = medium, 3 = dense), mean libido score (0 = no libido, to 4 = high libido) and mean pooled attractiveness rank ± SD (1 = very attractive, to 7 = not attractive) with and without blinds in oestrous and dioestrous tests of the seven stallions used in the study.

Stallion	Age	Body	Body	Body	Mane	Mean	Mean attractiveness rank^1^	
	(years)	height	weight	condition	density	libido	(± SD)	
		(cm)	(kg)	score	score	score	Oestrus	Dioestrus
A	19	155	551	5	1	3.00	5.00 (1.54)	4.09 (1.62)
B	18	157	582	5	3	1.25	3.66 (1.27)	3.51 (1.56)
C	16	158	560	4	2	4.00	3.08 (1.15)	3.59 (1.32)
D^2^	15	159	575	5	1	1.50	5.49 (1.51)	3.37 (1.85)
E	9	157	540	4	3	1.00	5.21 (1.38)	3.93 (1.21)
F	6	155	520	3	2	2.50	2.28 (0.99)	3.97 (1.60)
G	5	154	505	3	2	2.00	3.18 (1.32)	4.59 (0.97)

1 three of 19 mares were unavailable for the tests without blinds.

^2^ had to be euthanized towards end of study because of acute colic and was unavailable for 6 tests in oestrus and 7 in dioestrus, respectively.

**Fig 3:**
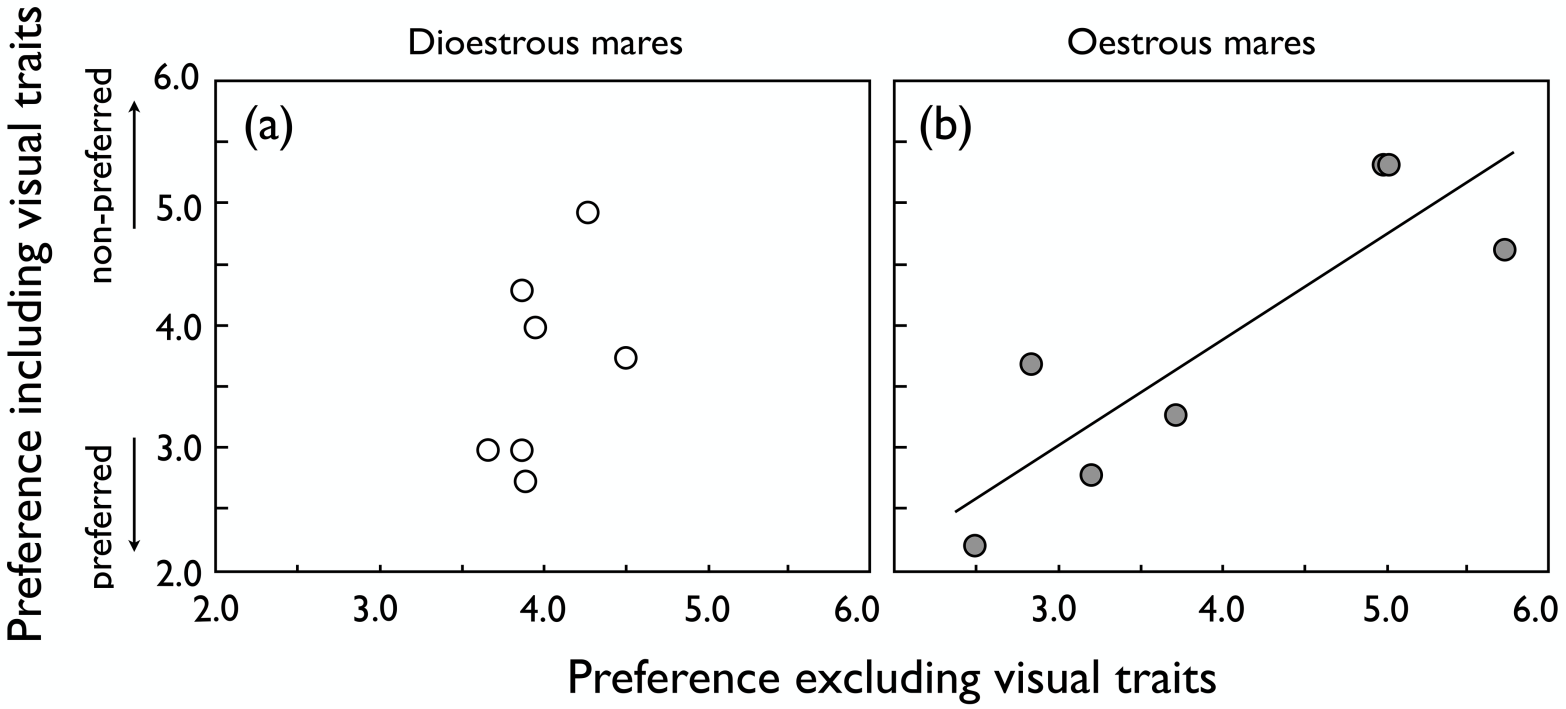
Repeatability of mean preference scores. Repeatability of mean preference scores with or without blinds that prevented visual contact in (a) dioestrous mares (r_s_ = 0.39, *P* = 0.39) and (b) oestrous mares (r_s_ = 0.78, *P* = 0.04; regression line drawn for illustration).

**Fig 4:**
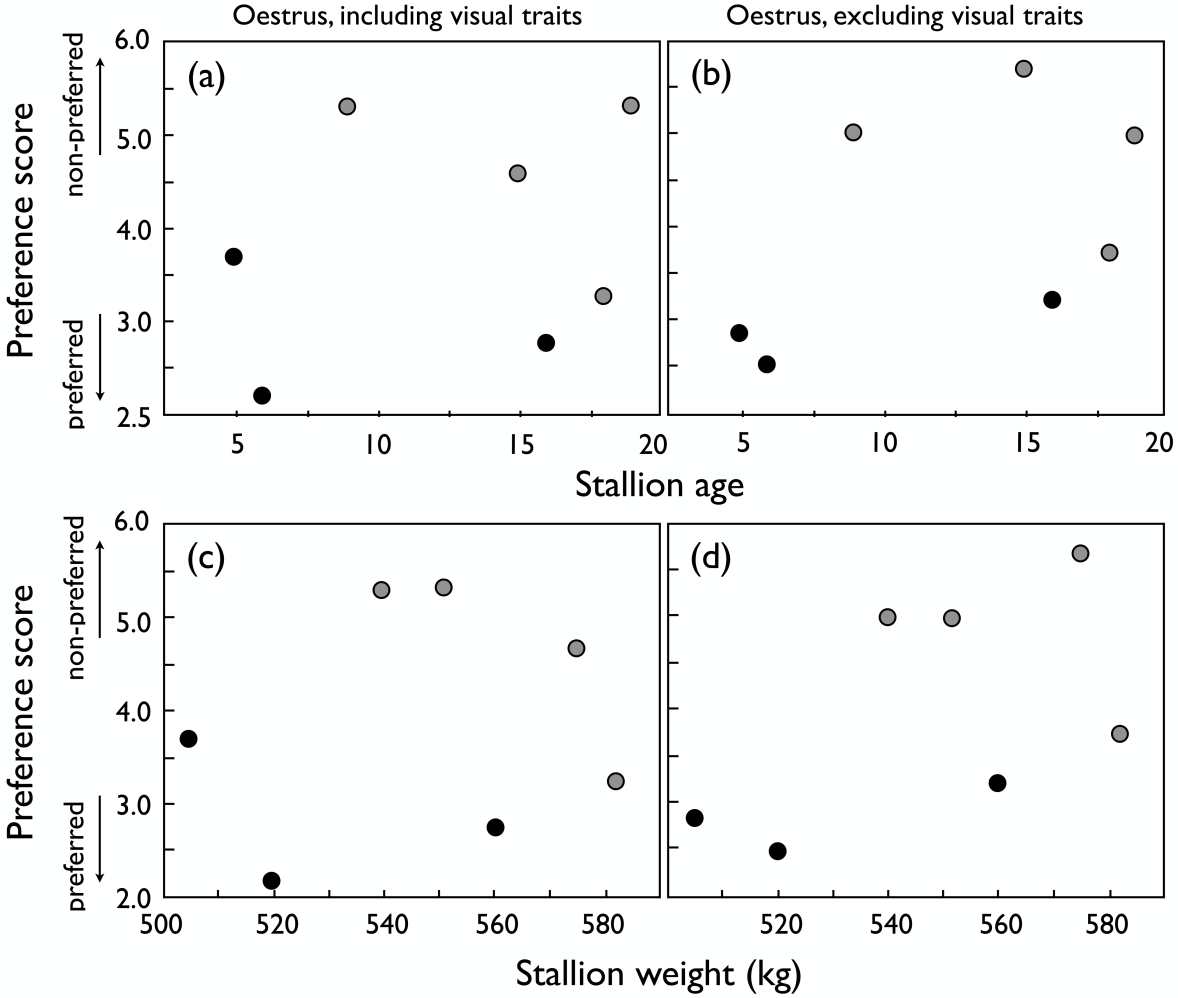
Preferences of oestrous mares. Mean preference scores (1 = very attractive to 7 = not attractive) of oestrous mares for stallions of different age when (a) visual contact was possible (r_s_ = 0.25, *P* = 0.59), and (b) visual contact was prevented (r_s_ = 0.50, *P* = 0.25). The corresponding correlations to stallion weight are given in panels (c) (r_s_ = 0.02, *P* = 0.97) and (d) (r_s_ = 0.56, *P* = 0.19), respectively. Stallions that showed signs of a higher libido during routine sperm collection are indicated with black symbols.

While the preference scores of dioestrous females with and without blinds were not significantly correlated (Fig 3A), oestrous females showed consistent preferences with and without blinds, i.e. their preferences were mainly based on non-visual traits (Fig 3B). Male age (Fig 4A and 4B) and size (Fig 4C and 4D) did not significantly influence the preference of oestrous females regardless of whether visual contact was possible or not. Accordingly, the preferences of dioestrous and oestrous females were not correlated when visual contact was allowed for (r_s_ = 0.04, *P* = 0.94), but they were also not correlated when visual contact was prevented (r_s_ = -0.04, *P* = 0.94).

There was no significant change in libido of the stallions over time (mixed effect model on libido score, with week as fixed factor and stallion identity as random factor, effect of week: F = 1.3, d.f. = 3,12.3 p = 0.30), but the individual males differed significantly in their libido score as determined during the four routine semen collections: whereas sperm could be obtained from six of the seven males, only three males always ejaculated on the dummy while the other males appeared to need an additional stimulus at all times. These three males tended to be preferred by oestrous females (Fig 4). Semen of one stallion could not be collected due to unwillingness to mount the dummy or the mare. In addition, while four stallions showed high libido whilst in contact with the mares throughout the experimental period, one of the two least favoured stallions became progressively aggressive and the other noticeably reluctant, respectively.

## Discussion

We found that female preferences for visual or non-visual male traits depend on the stage of their oestrous cycle. Visual traits were important during dioestrus but seemed to play a minor role during oestrus. Dioestrous mares showed a strong preference for the proximity of older and larger stallions based on visual traits alone. When visual contact was prevented and only vocal or olfactory contact was allowed for, female discrimination of stallions seemed no longer based on size or age.

Female preference for older and/or larger males may often be expected, especially in polygynous species, and has been found in other mammals, too (e.g. the African elephant, *Loxodonta africana* [57]). Under feral conditions, young stallions are unlikely to become harem stallions [38], i.e. traits that signal age could be interpreted by mares as traits of likely harem stallions. If age is positively correlated with size, as observed in the present study, female preference for old and large males could correspond to the likely outcome of male-male agonistic interactions (i.e. the type of interactions that we tried to control for with our experimental set up). Females may then be more likely to obtain potential direct benefits when choosing older or larger males if they hold a higher social status [58] and be able to provide more paternal support for offspring [59–61] than younger males. However, even if age and size were not correlated, age by itself could signal genetic quality, i.e. high genetic quality having positively influenced the survivability of older males [62,63].

In contrast, we found that females in oestrus showed preferences that were mainly based on non-visual traits (because their preferences were consistent when tested with and without blinds) and that were not significantly influenced by male age and size. The relative importance of olfactory versus vocal traits remains unsolved here. Vocal displays have been shown to affect female hormone levels and to influence ovulation in several mammals (red deer, *Cervus elaphus* [64,65]; fallow deer, *Dama dama* [66]; grey mouse lemur, *Microcebus murinus* [67]). In our analyses, the vocal influences on the mares’ decisions could not be analysed and would have to be studied in a different experimental design, i.e. a design that controls for potentially confounding factors such as male-male vocal interactions or olfactory signals. On the other hand, female preferences have often been found to be influenced by olfactory displays in mammals ([68,69]; reviewed by Clutton-Brock and McAuliffe [5]). The scent of a male may reflect his condition and how much it suffers from infections [70,71], allowing females to avoid infection or potentially get good genes for the common offspring if resistances to infection are heritable. In our study, however, an effect of pathogens seems unlikely as all males were dewormed prior to the experiments and showed no signs of infections. Odours also reveal information about various important genes, including the highly polymorphic MHC genes that have been shown to influence odours and mate preferences in many vertebrates [47,48]. Such MHC-linked preferences seem to generally promote MHC heterozygosity among offspring because MHC-dissimilar types are typically preferred. Indeed, when serotyping the mares and stallions of the present study for their MHC types, we found also that mares preferred the odours of MHC-dissimilar stallions, i.e. of stallions who shared no MHC allele with the mare [50]. These MHC effects were statistically significant when tested in dioestrous mares, but not so when tested during oestrus, suggesting that non-MHC linked male characteristics may, to some degree, overshadow MHC effects during oestrus [50]. Larger sample sizes would then be necessary to demonstrate MHC effects also during oestrus. Indeed, a recent study based on 191 mares [40] demonstrated that MHC-linked social signalling during oestrus had a significant effect on female fertility, i.e. it affected cryptic female choice. Potentially confounding effects of semen quality or embryo genetics were experimentally controlled via instrumental insemination.

The differences of mares’ preferences for certain stallion characteristics that we observed here may reflect the varying physiological and context-dependant requirements of females in this species. Preferences may hence be displayed dependant on relative importance of male traits at a particular time and status. Indeed, beside MHC-associated preferences, females in dioestrus, i.e. most of the time in an oestrous cycle, during entire gestation and partially whilst dispersal activities, may exhibit a higher demand for a dominant and experienced harem male, e.g. when protection, herd cohesion and paternal care for the offspring provided by a male may be more attractive than other male traits. In contrast, oestrous females may show different preferences during the relatively short state of oestrus, dispersal and copulation time, and certain genetic male characteristics may then become more attractive than other traits. Indeed, it has often been observed that not all foals of a harem originated from the dominant harem stallion. Harem stallions either tolerate or are not able to prevent copulations between mares of his harem and other stallions, especially if the mare is a close kin [72,73]. Our observations suggest that male age, size, and probably other visual traits play no decisive role in mate preference of oestrous mares. It remains to be elucidated in further studies what additional male characteristics might influence mares’ preferences in oestrus.

The experimental procedure used in this present study proofed to be useful for studying female preferences in horses. We observed much variance in female behaviour within the experimental situation, as expected from previous studies on female preferences [74]. Intra-sexual competition among the males was reduced by our study design with stallions being separated in boxes, but it is still possible that they influenced each other by, for example, sounds and odours. It is also possible that some types of interactions between females and males affected male behaviour that in turn influenced female preferences. We observed during the experimental period that the males significantly differed in their libido. Higher libido was displayed by the generally more preferred stallions, which may have been a consequence of female interest, or which may have positively contributed to it.

We did not assess dominance ranking in the present study. We suggest that the possible effects of male dominance should be assessed in further studies. A strong effect of social status has already been suggested in domestic goats, *Capra hircus* [75] or free-ranging dogs, *Canis familiaris* [76,77] where highest-ranked males were perceived as generally more attractive. Indeed, over the course of the experiment there seemed to be a change of behaviour towards being reluctant or aggressive in the two least preferred stallions.

Some theories of sexual selection predict little variation in female preferences, especially theories that are based on handicapping signals (e.g. [78,79]). However, mammals often use non-handicapping traits that can still reveal honest information about various important mate characteristics [80] and whose attractiveness may depend on individual female characteristics [81,82]. Moreover, life history theory predicts that female preferences to be often context dependent [1]. We would therefore predict variance in female preferences when tested under different conditions. Here we found that female preferences in horses depend both on the mares’ oestrous cycle and whether or not visual traits are available.

## Supporting information

S1 Table Contact times (seconds) of 19 mares with 7 stallions (#1 - #7) tested in 4 types of tests (oestrus or dioestrus with or without blinds) and 6 test rounds each. The highest contact time per round is marked in bold. The respective stallion was then removed before the next round. If a mare showed no interest in any of the stallions (all contact times = 0), the test was considered as finished. NA = stallion or mare was not available for the respective test.
(DOCX)

S2 Table Completed and non-completed test series (*N*) per test type in 7 stallions and 19 mares, contacted stallions per test round (%), mean contact time ± SD (s), mean number of oestrous/ investigative/ affiliative and agonistic behaviours ± SD (*N*) of the mares per contacted stallion. (DOCX)
