# Supplementary material for: Cycle-specific female preferences for visual and non-visual cues in the horse (Equus caballus)
Source: PLoS One. 2018 Feb 21;13(2):e0191845. doi: 10.1371/journal.pone.0191845 (PMC5842875; doi:10.1371/journal.pone.0191845)
Supplement: S1 Table — The highest contact time per round is marked in bold. The respective stallion was then removed before the next round. If a mare showed no interest in any of the stallions (all contact times = 0), the test was considered as finished. NA = stallion or mare was not available for the respective test. (DOCX) [file pone.0191845.s001.docx]

**Table S1.** Contact times (seconds) of 19 mares with 7 stallions (#1 - #7) tested in 4 types of tests (oestrus or dioestrus with or without blinds) and 6 test rounds each. The highest contact time per round is marked in bold. The respective stallion was then removed before the next round. If a mare showed no interest in any of the stallions (all contact times = 0), the test was considered as finished. NA = stallion or mare was not available for the respective test.

| **Mare** | **Test** | **Round** | **Contact time to stallion** | | | | | | |
| --- | --- | --- | --- | --- | --- | --- | --- | --- | --- |
|  |  |  | **#1** | **#2** | **#3** | **#4** | **#5** | **#6** | **#7** |
| 1 | Oestrus with blinds | 1 | 14 | 22 | 6 | 65 | 30 | **267** | 0 |
|  |  | 2 | 18 | 24 | 83 | 15 | 151 |  | **187** |
|  |  | 3 | 5 | 13 | 36 | **187** | 182 |  |  |
|  |  | 4 | 11 | 67 | **227** |  | 41 |  |  |
|  |  | 5 | 15 | **25** |  |  | 20 |  |  |
|  |  | 6 | **106** |  |  |  | 42 |  |  |
|  | Dioestrus with blinds | 1 | 9 | 1 | 0 | 0 | 0 | **306** | 0 |
|  |  | 2 | 0 | 10 | 3 | 0 | **148** |  | 0 |
|  |  | 3 | 48 | **119** | 15 | 22 |  |  | 0 |
|  |  | 4 | **81** |  | 0 | 0 |  |  | 25 |
|  |  | 5 |  |  | 12 | 11 |  |  | **205** |
|  |  | 6 |  |  | **21** | 0 |  |  |  |
|  | Oestrus without blinds | 1 | NA | NA | NA | NA | NA | NA | NA |
|  |  | 2 | NA | NA | NA | NA | NA | NA | NA |
|  |  | 3 | NA | NA | NA | NA | NA | NA | NA |
|  |  | 4 | NA | NA | NA | NA | NA | NA | NA |
|  |  | 5 | NA | NA | NA | NA | NA | NA | NA |
|  |  | 6 | NA | NA | NA | NA | NA | NA | NA |
|  | Dioestrus without blinds | 1 | NA | NA | NA | NA | NA | NA | NA |
|  |  | 2 | NA | NA | NA | NA | NA | NA | NA |
|  |  | 3 | NA | NA | NA | NA | NA | NA | NA |
|  |  | 4 | NA | NA | NA | NA | NA | NA | NA |
|  |  | 5 | NA | NA | NA | NA | NA | NA | NA |
|  |  | 6 | NA | NA | NA | NA | NA | NA | NA |
| 2 | Oestrus with blinds | 1 | 4 | 31 | 53 | 85 | 46 | **197** | 18 |
|  |  | 2 | 25 | **109** | 78 | 17 | 19 |  | 60 |
|  |  | 3 | 16 |  | 71 | 2 | 78 |  | **249** |
|  |  | 4 | 0 |  | **214** | 48 | 52 |  |  |
|  |  | 5 | 46 |  |  | 27 | **127** |  |  |
|  |  | 6 | **46** |  |  | 2 |  |  |  |
|  | Dioestrus with blinds | 1 | 0 | 10 | 38 | 9 | 0 | **103** | 10 |
|  |  | 2 | 5 | 7 | **106** | 81 | 0 |  | 9 |
|  |  | 3 | 0 | 78 |  | 0 | 7 |  | **92** |
|  |  | 4 | **59** | 26 |  | 11 | 17 |  |  |
|  |  | 5 |  | 21 |  | **72** | 4 |  |  |
|  |  | 2 |  | **520** |  |  | 0 |  |  |
|  | Oestrus without blinds | 1 | 25 | 113 | **211** | 6 | 32 | 38 | 19 |
|  |  | 2 | 16 | **192** |  | 8 | 13 | 92 | 58 |
|  |  | 3 | 35 |  |  | 16 | 13 | **207** | 19 |
|  |  | 4 | 35 |  |  | 30 | 44 |  | **126** |
|  |  | 5 | 61 |  |  | **212** | 138 |  |  |
|  |  | 6 | 69 |  |  |  | **217** |  |  |
|  | Dioestrus without blinds | 1 | 0 | **248** | 91 | 49 | 6 | 2 | 4 |
|  |  | 2 | **79** |  | 9 | 36 | 0 | 0 | 0 |
|  |  | 3 |  |  | **78** | 0 | 0 | 9 | 0 |
|  |  | 4 |  |  |  | 5 | **57** | 20 | 0 |
|  |  | 5 |  |  |  | **52** |  | 0 | 8 |
|  |  | 6 |  |  |  |  |  | 0 | **5** |
| 3 | Oestrus with blinds | 1 | 0 | 0 | **513** | 0 | 0 | 0 | 9 |
|  |  | 2 | 0 | 54 |  | 0 | 0 | 0 | **352** |
|  |  | 3 | **456** | 0 |  | 0 | 0 | 0 |  |
|  |  | 4 |  | 0 |  | 0 | 0 | **440** |  |
|  |  | 5 |  | **175** |  | 0 | 0 |  |  |
|  |  | 6 |  |  |  | 0 | **226** |  |  |
|  | Dioestrus with blinds | 1 | 0 | 0 | 0 | 0 | 0 | 0 | 0 |
|  |  | 2 |  |  |  |  |  |  |  |
|  |  | 3 |  |  |  |  |  |  |  |
|  |  | 4 |  |  |  |  |  |  |  |
|  |  | 5 |  |  |  |  |  |  |  |
|  |  | 6 |  |  |  |  |  |  |  |
|  | Oestrus without blinds | 1 | 0 | 257 | 0 | NA | 0 | 0 | **265** |
|  |  | 2 | 83 | 130 | 133 | NA | 39 | **155** |  |
|  |  | 3 | **202** | 68 | 178 | NA | 60 |  |  |
|  |  | 4 |  | **201** | 22 | NA | 127 |  |  |
|  |  | 5 |  |  | **244** | NA | 61 |  |  |
|  |  | 6 |  |  |  | NA |  |  |  |
|  | Dioestrus without blinds | 1 | 0 | **460** | 4 | 51 | 0 | 0 | 4 |
|  |  | 2 | 0 |  | 13 | **16** | 0 | 15 | 0 |
|  |  | 3 | 12 |  | 5 |  | 48 | 0 | **237** |
|  |  | 4 | 0 |  | 0 |  | **26** | 0 |  |
|  |  | 5 | 7 |  | 113 |  |  | **124** |  |
|  |  | 6 | 12 |  | **80** |  |  |  |  |
| 4 | Oestrus with blinds | 1 | 18 | 0 | 123 | 39 | 0 | 0 | **246** |
|  |  | 2 | 0 | 0 | **104** | 67 | 93 | 97 |  |
|  |  | 3 | 0 | **200** |  | 27 | 34 | 130 |  |
|  |  | 4 | 0 |  |  | **138** | 82 | 5 |  |
|  |  | 5 | 0 |  |  |  | 13 | **212** |  |
|  |  | 6 | 41 |  |  |  | **243** |  |  |
|  | Dioestrus with blinds | 1 | 19 | 0 | 0 | **44** | 0 | 0 | 6 |
|  |  | 2 | **187** | 0 | 0 |  | 0 | 0 | 0 |
|  |  | 3 |  | 0 | 0 |  | 0 | 0 | 0 |
|  |  | 4 |  |  |  |  |  |  |  |
|  |  | 5 |  |  |  |  |  |  |  |
|  |  | 6 |  |  |  |  |  |  |  |
|  | Oestrus without blinds | 1 | 33 | 126 | 28 | NA | 0 | **164** | 92 |
|  |  | 2 | 38 | 28 | 77 | NA | 0 |  | **257** |
|  |  | 3 | **133** | 24 | 125 | NA | 20 |  |  |
|  |  | 4 |  | 67 | **159** | NA | 0 |  |  |
|  |  | 5 |  | **235** |  | NA | 26 |  |  |
|  |  | 6 |  |  |  | NA |  |  |  |
|  | Dioestrus without blinds | 1 | 0 | 0 | 0 | NA | 3 | **138** | 0 |
|  |  | 2 | 0 | 3 | **19** | NA | 5 |  | 0 |
|  |  | 3 | 3 | **5** |  | NA | 0 |  | 0 |
|  |  | 4 | **20** |  |  | NA | 0 |  | 0 |
|  |  | 5 |  |  |  | NA | **2** |  | 0 |
|  |  | 6 |  |  |  | NA |  |  |  |
| 5 | Oestrus with blinds | 1 | 0 | 0 | **144** | 0 | 31 | 20 | 115 |
|  |  | 2 | 0 | 23 |  | 0 | **140** | 28 | 0 |
|  |  | 3 | 28 | **89** |  | 55 |  | 0 | 0 |
|  |  | 4 | 0 |  |  | 0 |  | 0 | 0 |
|  |  | 5 |  |  |  |  |  |  |  |
|  |  | 6 |  |  |  |  |  |  |  |
|  | Dioestrus with blinds | 1 | **253** | 0 | 0 | 0 | 0 | 0 | 0 |
|  |  | 2 |  | 0 | 0 | 0 | 0 | **5** | 0 |
|  |  | 3 |  | 0 | 0 | 0 | 0 |  | 0 |
|  |  | 4 |  |  |  |  |  |  |  |
|  |  | 5 |  |  |  |  |  |  |  |
|  |  | 6 |  |  |  |  |  |  |  |
|  | Oestrus without blinds | 1 | 47 | 9 | **159** | 104 | 50 | 67 | 54 |
|  |  | 2 | 50 | 52 |  | **116** | 9 | 81 | 71 |
|  |  | 3 | 14 | 95 |  |  | 21 | **200** | 50 |
|  |  | 4 | 61 | **225** |  |  | 91 |  | 47 |
|  |  | 5 | **204** |  |  |  | 29 |  | 186 |
|  |  | 6 |  |  |  |  | 143 |  | **223** |
|  | Dioestrus without blinds | 1 | 0 | 0 | 9 | **221** | 6 | 0 | 0 |
|  |  | 2 | 30 | 0 | 1 |  | **46** | 0 | 0 |
|  |  | 3 | 3 | 5 | **20** |  |  | 0 | 11 |
|  |  | 4 | 0 | **5** |  |  |  | 0 | 0 |
|  |  | 5 | 0 |  |  |  |  | 0 | **6** |
|  |  | 6 | **392** |  |  |  |  | 24 |  |
| 6 | Oestrus with blinds | 1 | 24 | 19 | 43 | 0 | 0 | 54 | **211** |
|  |  | 2 | 55 | 68 | 22 | 5 | 0 | **285** |  |
|  |  | 3 | 149 | 40 | **176** | 52 | 0 |  |  |
|  |  | 4 | 44 | **124** |  | 99 | 47 |  |  |
|  |  | 5 | 97 |  |  | **127** | 33 |  |  |
|  |  | 6 | **83** |  |  |  | 68 |  |  |
|  | Dioestrus with blinds | 1 | 0 | 2 | 5 | 0 | 0 | 2 | 4 |
|  |  | 2 | 14 | 15 |  | 1 | 10 | 5 | 0 |
|  |  | 3 | 18 |  |  | 89 | 63 | 7 | 0 |
|  |  | 4 | 0 |  |  |  | 480 | 0 | 0 |
|  |  | 5 | 0 |  |  |  |  | 0 | 0 |
|  |  | 6 |  |  |  |  |  |  |  |
|  | Oestrus without blinds | 1 | 33 | 70 | 5 | **123** | 73 | 42 | 35 |
|  |  | 2 | 5 | 134 | **185** |  | 0 | 7 | 3 |
|  |  | 3 | 0 | 73 |  |  | 83 | **197** | 95 |
|  |  | 4 | 10 | 100 |  |  | **189** |  | 68 |
|  |  | 5 | 17 | 90 |  |  |  |  | **137** |
|  |  | 6 | 8 | **402** |  |  |  |  |  |
|  | Dioestrus without blinds | 1 | 5 | 97 | 8 | **100** | 0 | 0 | 2 |
|  |  | 2 | 0 | 8 | **29** |  | 13 | 0 | 2 |
|  |  | 3 | 0 | 0 |  |  | 4 | 3 | **20** |
|  |  | 4 | 0 | 0 |  |  | **3** | 0 |  |
|  |  | 5 | 0 | 0 |  |  |  | 0 |  |
|  |  | 6 |  |  |  |  |  |  |  |
| 7 | Oestrus with blinds | 1 | 14 | 0 | 11 | 0 | 0 | **67** | 25 |
|  |  | 2 | 0 | 0 | 0 | **5** | 0 |  | 0 |
|  |  | 3 | 0 | 0 | 101 |  | 0 |  | **207** |
|  |  | 4 | 7 | **55** | 0 |  | 0 |  |  |
|  |  | 5 | 0 |  | **82** |  | 0 |  |  |
|  |  | 6 | 83 |  |  |  | **123** |  |  |
|  | Dioestrus with blinds | 1 | 3 | **9** | 7 | 1 | 0 | 0 | 6 |
|  |  | 2 | 4 |  | **20** | 0 | 0 | 16 | 0 |
|  |  | 3 | **33** |  |  | 12 | 0 | 17 | 5 |
|  |  | 4 |  |  |  | 0 | 3 | 8 | **11** |
|  |  | 5 |  |  |  | 0 | 0 | **14** |  |
|  |  | 6 |  |  |  | 3 | **42** |  |  |
|  | Oestrus without blinds | 1 | 13 | **133** | 73 | 6 | 28 | 117 | 43 |
|  |  | 2 | 0 |  | 102 | 16 | 68 | **244** | 19 |
|  |  | 3 | 0 |  | 114 | 0 | 80 |  | **261** |
|  |  | 4 | 57 |  | 82 | **172** | 44 |  |  |
|  |  | 5 | 60 |  | 95 |  | **180** |  |  |
|  |  | 6 | 21 |  | **178** |  |  |  |  |
|  | Dioestrus without blinds | 1 | 20 | **59** | 3 | 7 | 0 | 4 | 33 |
|  |  | 2 | **340** |  | 26 | 0 | 0 | 22 | 0 |
|  |  | 3 |  |  | **34** | 48 | 15 | 33 | 59 |
|  |  | 4 |  |  |  | **20** | 0 | 12 | 0 |
|  |  | 5 |  |  |  |  | 0 | **23** | 0 |
|  |  | 6 |  |  |  |  | **49** |  | 15 |
| 8 | Oestrus with blinds | 1 | 0 | 74 | 6 | 7 | 128 | 65 | **218** |
|  |  | 2 | 0 | 36 | 15 | 56 | 57 | **355** |  |
|  |  | 3 | **144** | 135 | 28 | 61 | 90 |  |  |
|  |  | 4 |  | 45 | **214** | 64 | 62 |  |  |
|  |  | 5 |  | **365** |  | 0 | 82 |  |  |
|  |  | 6 |  |  |  | **283** | 0 |  |  |
|  | Dioestrus with blinds | 1 | 0 | 46 | 0 | 3 | **75** | 0 | 65 |
|  |  | 2 | 4 | 84 | 0 | 49 |  | 38 | **176** |
|  |  | 3 | 9 | **165** | 113 | 0 |  | 0 |  |
|  |  | 4 | 0 |  | 0 | **1** |  | 0 |  |
|  |  | 5 | 0 |  | **50** |  |  | 0 |  |
|  |  | 6 | 0 |  |  |  |  | 0 |  |
|  | Oestrus without blinds | 1 | 0 | 64 | 36 | 15 | 27 | **241** | 56 |
|  |  | 2 | 0 | 34 | **241** | 5 | 22 |  | 172 |
|  |  | 3 | 0 | 38 |  | 8 | 4 |  | **448** |
|  |  | 4 | 0 | **188** |  | 141 | 151 |  |  |
|  |  | 5 | 40 |  |  | **205** | 199 |  |  |
|  |  | 6 | 96 |  |  |  | **220** |  |  |
|  | Dioestrus without blinds | 1 | 0 | 0 | **120** | 76 | 31 | 0 | 33 |
|  |  | 2 | 8 | **291** |  | 7 | 1 | 59 | 0 |
|  |  | 3 | 0 |  |  | 41 | 12 | 0 | **106** |
|  |  | 4 | 0 |  |  | **368** | 130 | 47 |  |
|  |  | 5 | 0 |  |  |  | **102** | 55 |  |
|  |  | 6 | 0 |  |  |  |  | 0 |  |
| 9 | Oestrus with blinds | 1 | **25** | 0 | 3 | 0 | 3 | 17 | 4 |
|  |  | 2 |  | 0 | 11 | 0 | 5 | **17** | 0 |
|  |  | 3 |  | 0 | **12** | 0 | 11 |  | 0 |
|  |  | 4 |  | **23** |  | 6 | 0 |  | 18 |
|  |  | 5 |  |  |  | 0 | 8 |  | **9** |
|  |  | 6 |  |  |  | **13** | 0 |  |  |
|  | Dioestrus with blinds | 1 | 0 | **17** | 0 | 10 | 0 | 9 | 0 |
|  |  | 2 | 4 |  | 0 | **6** | 0 | 5 | 5 |
|  |  | 3 | 2 |  | 0 |  | 0 | **7** | 0 |
|  |  | 4 | 5 |  | 4 |  | **14** |  | 0 |
|  |  | 5 | 0 |  | **14** |  |  |  | 0 |
|  |  | 6 | 10 |  |  |  |  |  | **20** |
|  | Oestrus without blinds | 1 | 36 | 117 | 74 | 6 | 18 | **146** | 22 |
|  |  | 2 | 20 | 69 | 64 | 0 | 25 |  | **257** |
|  |  | 3 | 62 | 50 | **244** | 0 | 11 |  |  |
|  |  | 4 | 59 | **197** |  | 29 | 39 |  |  |
|  |  | 5 | **215** |  |  | 26 | 62 |  |  |
|  |  | 6 |  |  |  | 66 | **176** |  |  |
|  | Dioestrus without blinds | 1 | 0 | **76** | 43 | NA | 5 | 0 | 0 |
|  |  | 2 | 0 |  | **47** | NA | 11 | 2 | 0 |
|  |  | 3 | 0 |  |  | NA | 4 | **7** | 0 |
|  |  | 4 | 16 |  |  | NA | 19 |  | **21** |
|  |  | 5 | **19** |  |  | NA | 0 |  |  |
|  |  | 6 |  |  |  | NA |  |  |  |
| 10 | Oestrus with blinds | 1 | 10 | 0 | 58 | 60 | 0 | **157** | 6 |
|  |  | 2 | 40 | 17 | 47 | 42 | 70 |  | **219** |
|  |  | 3 | **46** | 41 | 43 | 23 | 33 |  |  |
|  |  | 4 |  | **187** | 22 | 0 | 0 |  |  |
|  |  | 5 |  |  | 23 | **46** | 0 |  |  |
|  |  | 6 |  |  | **231** |  | 0 |  |  |
|  | Dioestrus with blinds | 1 | 0 | 19 | 12 | 0 | 0 | 7 | **83** |
|  |  | 2 | 0 | **21** | 0 | 0 | 0 | 0 |  |
|  |  | 3 | 0 |  | **15** | 0 | 0 | 0 |  |
|  |  | 4 | 0 |  |  | **7** | 0 | 0 |  |
|  |  | 5 | 0 |  |  |  | 0 | 0 |  |
|  |  | 6 |  |  |  |  |  |  |  |
|  | Oestrus without blinds | 1 | 17 | **171** | 96 | 11 | 12 | 162 | 50 |
|  |  | 2 | 10 |  | 36 | 33 | 5 | **276** | 50 |
|  |  | 3 | 19 |  | **231** | 19 | 11 |  | 24 |
|  |  | 4 | 7 |  |  | 24 | 58 |  | **235** |
|  |  | 5 | 17 |  |  | 69 | **113** |  |  |
|  |  | 6 | 33 |  |  | **138** |  |  |  |
|  | Dioestrus without blinds | 1 | 4 | **101** | 21 | 15 | 45 | 92 | 36 |
|  |  | 2 | 29 |  | 0 | 8 | **35** | 37 | 10 |
|  |  | 3 | 17 |  | 18 | **126** |  | 43 | 0 |
|  |  | 4 | 0 |  | **72** |  |  | 52 | 19 |
|  |  | 5 | 15 |  |  |  |  | **64** | 5 |
|  |  | 6 | 0 |  |  |  |  |  | **4** |
| 11 | Oestrus with blinds | 1 | **181** | 3 | 11 | 0 | 17 | 81 | 38 |
|  |  | 2 |  | 30 | 0 | 2 | 12 | **110** | 0 |
|  |  | 3 |  | 21 | **27** | 22 | 19 |  | 10 |
|  |  | 4 |  | 26 |  | 14 | 0 |  | **28** |
|  |  | 5 |  | **48** |  | 0 | 10 |  |  |
|  |  | 6 |  |  |  | **153** | 0 |  |  |
|  | Dioestrus with blinds | 1 | **12** | 0 | 9 | 0 | 0 | 0 | 6 |
|  |  | 2 |  | 0 | 21 | **22** | 5 | 0 | 0 |
|  |  | 3 |  | 0 | **2** |  | 0 | 0 | 0 |
|  |  | 4 |  | 0 |  |  | 0 | 0 | 0 |
|  |  | 5 |  |  |  |  |  |  |  |
|  |  | 6 |  |  |  |  |  |  |  |
|  | Oestrus without blinds | 1 | NA | NA | NA | NA | NA | NA | NA |
|  |  | 2 | NA | NA | NA | NA | NA | NA | NA |
|  |  | 3 | NA | NA | NA | NA | NA | NA | NA |
|  |  | 4 | NA | NA | NA | NA | NA | NA | NA |
|  |  | 5 | NA | NA | NA | NA | NA | NA | NA |
|  |  | 6 | NA | NA | NA | NA | NA | NA | NA |
|  | Dioestrus without blinds | 1 | NA | NA | NA | NA | NA | NA | NA |
|  |  | 2 | NA | NA | NA | NA | NA | NA | NA |
|  |  | 3 | NA | NA | NA | NA | NA | NA | NA |
|  |  | 4 | NA | NA | NA | NA | NA | NA | NA |
|  |  | 5 | NA | NA | NA | NA | NA | NA | NA |
|  |  | 6 | NA | NA | NA | NA | NA | NA | NA |
| 12 | Oestrus with blinds | 1 | 0 | 17 | 49 | 0 | **85** | 0 | 60 |
|  |  | 2 | 0 | **101** | 0 | 0 |  | 0 | 0 |
|  |  | 3 | 0 |  | 0 | 0 |  | 0 | **188** |
|  |  | 4 | 0 |  | **218** | 0 |  | 0 |  |
|  |  | 5 | 15 |  |  | 0 |  | **159** |  |
|  |  | 6 | 0 |  |  | **434** |  |  |  |
|  | Dioestrus with blinds | 1 | 0 | 0 | 2 | 28 | **172** | 0 | 12 |
|  |  | 2 | **14** | 2 | 0 | 0 |  | 5 | 0 |
|  |  | 3 |  | 0 | 0 | **6** |  | 0 | 0 |
|  |  | 4 |  | 0 | 0 |  |  | 0 | **34** |
|  |  | 5 |  | 0 | **7** |  |  | 0 |  |
|  |  | 6 |  | **57** |  |  |  | 0 |  |
|  | Oestrus without blinds | 1 | 115 | **167** | 10 | 45 | 7 | 10 | 30 |
|  |  | 2 | 59 |  | **114** | 0 | 101 | 0 | 0 |
|  |  | 3 | 6 |  |  | 0 | 0 | **263** | 0 |
|  |  | 4 | 8 |  |  | 12 | 27 |  | **39** |
|  |  | 5 | 16 |  |  | 22 | **432** |  |  |
|  |  | 6 | **2** |  |  | 0 |  |  |  |
|  | Dioestrus without blinds | 1 | **144** | 115 | 0 | 91 | 22 | 4 | 0 |
|  |  | 2 |  | 0 | 0 | **177** | 0 | 0 | 0 |
|  |  | 3 |  | **71** | 0 |  | 0 | 3 | 4 |
|  |  | 4 |  |  | 0 |  | **4** | 0 | 0 |
|  |  | 5 |  |  | 0 |  |  | 0 | 0 |
|  |  | 6 |  |  |  |  |  |  |  |
| 13 | Oestrus with blinds | 1 | 0 | **383** | 0 | 9 | 164 | 0 | 0 |
|  |  | 2 | 0 |  | 0 | 0 | 0 | **420** | 0 |
|  |  | 3 | 0 |  | 173 | 0 | 0 |  | **404** |
|  |  | 4 | 98 |  | 70 | 0 | **318** |  |  |
|  |  | 5 | 6 |  | **195** | 72 |  |  |  |
|  |  | 6 | **85** |  |  | 71 |  |  |  |
|  | Dioestrus with blinds | 1 | 0 | 0 | 23 | 0 | **387** | 0 | 62 |
|  |  | 2 | 225 | 0 | 4 | 0 |  | **258** | 27 |
|  |  | 3 | 0 | 3 | 0 | 17 |  |  | **168** |
|  |  | 4 | 0 | 0 | **9** | 0 |  |  |  |
|  |  | 5 | **21** | 0 |  | 0 |  |  |  |
|  |  | 6 |  | **45** |  | 0 |  |  |  |
|  | Oestrus without blinds | 1 | 23 | 3 | **168** | 15 | 13 | 68 | 27 |
|  |  | 2 | 24 | 57 |  | 0 | **135** | 94 | 103 |
|  |  | 3 | 0 | 0 |  | 0 |  | 233 | **338** |
|  |  | 4 | 3 | 58 |  | 10 |  | **446** |  |
|  |  | 5 | 79 | **237** |  | 23 |  |  |  |
|  |  | 6 | 36 |  |  | **223** |  |  |  |
|  | Dioestrus without blinds | 1 | 16 | 101 | **131** | NA | 109 | 58 | 0 |
|  |  | 2 | 12 | 47 |  | NA | 41 | **74** | 0 |
|  |  | 3 | 28 | **45** |  | NA | 15 |  | 8 |
|  |  | 4 | **264** |  |  | NA | 0 |  | 75 |
|  |  | 5 |  |  |  | NA | 19 |  | **92** |
|  |  | 6 |  |  |  | NA |  |  |  |
| 14 | Oestrus with blinds | 1 | 0 | 0 | 20 | 0 | 0 | **275** | 78 |
|  |  | 2 | 190 | 59 | 0 | 16 | 0 |  | **279** |
|  |  | 3 | **223** | 134 | 31 | 27 | 0 |  |  |
|  |  | 4 |  | 31 | 30 | 124 | **151** |  |  |
|  |  | 5 |  | 67 | **97** | 172 |  |  |  |
|  |  | 6 |  | **150** |  | 68 |  |  |  |
|  | Dioestrus with blinds | 1 | 0 | 11 | 35 | **90** | 0 | 49 | 42 |
|  |  | 2 | 10 | 0 | 0 |  | 0 | **36** | 22 |
|  |  | 3 | 22 | 16 | 0 |  | **77** |  | 10 |
|  |  | 4 | 13 | 0 | 0 |  |  |  | **14** |
|  |  | 5 | **14** | 9 | 0 |  |  |  |  |
|  |  | 6 |  | 0 | **49** |  |  |  |  |
|  | Oestrus without blinds | 1 | NA | NA | NA | NA | NA | NA | NA |
|  |  | 2 | NA | NA | NA | NA | NA | NA | NA |
|  |  | 3 | NA | NA | NA | NA | NA | NA | NA |
|  |  | 4 | NA | NA | NA | NA | NA | NA | NA |
|  |  | 5 | NA | NA | NA | NA | NA | NA | NA |
|  |  | 6 | NA | NA | NA | NA | NA | NA | NA |
|  | Dioestrus without blinds | 1 | NA | NA | NA | NA | NA | NA | NA |
|  |  | 2 | NA | NA | NA | NA | NA | NA | NA |
|  |  | 3 | NA | NA | NA | NA | NA | NA | NA |
|  |  | 4 | NA | NA | NA | NA | NA | NA | NA |
|  |  | 5 | NA | NA | NA | NA | NA | NA | NA |
|  |  | 6 | NA | NA | NA | NA | NA | NA | NA |
| 15 | Oestrus with blinds | 1 | 7 | 0 | 20 | 17 | **115** | 0 | 52 |
|  |  | 2 | 0 | 191 | 0 | 68 |  | 69 | **168** |
|  |  | 3 | 18 | **191** | 0 | 7 |  | 129 |  |
|  |  | 4 | 21 |  | **370** | 0 |  | 36 |  |
|  |  | 5 | 134 |  |  | 62 |  | **247** |  |
|  |  | 6 | **430** |  |  | 0 |  |  |  |
|  | Dioestrus with blinds | 1 | 0 | 0 | 0 | 0 | 0 | **12** | 0 |
|  |  | 2 | 0 | 0 | 0 | **3** | 0 |  | 0 |
|  |  | 3 | 0 | 0 | 0 |  | 0 |  | 0 |
|  |  | 4 |  |  |  |  |  |  |  |
|  |  | 5 |  |  |  |  |  |  |  |
|  |  | 6 |  |  |  |  |  |  |  |
|  | Oestrus without blinds | 1 | 4 | 34 | 184 | NA | 18 | **233** | 22 |
|  |  | 2 | 0 | 183 | **269** | NA | 56 |  | 25 |
|  |  | 3 | 128 | 127 |  | NA | **152** |  | 85 |
|  |  | 4 | **207** | 96 |  | NA |  |  | 148 |
|  |  | 5 |  | **182** |  | NA |  |  | 57 |
|  |  | 6 |  |  |  | NA |  |  |  |
|  | Dioestrus without blinds | 1 | **259** | 0 | 0 | NA | 0 | 0 | 0 |
|  |  | 2 |  | 0 | **53** | NA | 0 | 0 | 0 |
|  |  | 3 |  | 8 |  | NA | 0 | 55 | **125** |
|  |  | 4 |  | 0 |  | NA | 0 | **238** |  |
|  |  | 5 |  | 5 |  | NA | **301** |  |  |
|  |  | 6 |  |  |  | NA |  |  |  |
| 16 | Oestrus with blinds | 1 | 0 | 0 | **459** | 74 | 0 | 0 | 0 |
|  |  | 2 | 8 | **365** |  | 0 | 0 | 77 | 57 |
|  |  | 3 | 8 |  |  | 0 | 30 | **277** | 162 |
|  |  | 4 | 67 |  |  | 0 | 32 |  | **194** |
|  |  | 5 | 25 |  |  | 0 | **140** |  |  |
|  |  | 6 | **85** |  |  | 0 |  |  |  |
|  | Dioestrus with blinds | 1 | 0 | 0 | **22** | 0 | 0 | 0 | 2 |
|  |  | 2 | 0 | 0 |  | 0 | 0 | 0 | 0 |
|  |  | 3 |  |  |  |  |  |  |  |
|  |  | 4 |  |  |  |  |  |  |  |
|  |  | 5 |  |  |  |  |  |  |  |
|  |  | 6 |  |  |  |  |  |  |  |
|  | Oestrus without blinds | 1 | 52 | **142** | 39 | NA | 10 | 21 | 0 |
|  |  | 2 | 5 |  | **150** | NA | 42 | 0 | 9 |
|  |  | 3 | **412** |  |  | NA | 64 | 25 | 4 |
|  |  | 4 |  |  |  | NA | 86 | **205** | 14 |
|  |  | 5 |  |  |  | NA | 7 |  | **205** |
|  |  | 6 |  |  |  | NA |  |  |  |
|  | Dioestrus without blinds | 1 | **14** | 0 | 0 | NA | 2 | 0 | 9 |
|  |  | 2 |  | **414** | 0 | NA | 12 | 0 | 3 |
|  |  | 3 |  |  | 0 | NA | **1** | 0 | 0 |
|  |  | 4 |  |  | 0 | NA |  | 0 | **6** |
|  |  | 5 |  |  | 0 | NA |  | 0 |  |
|  |  | 6 |  |  |  | NA |  |  |  |
| 17 | Oestrus with blinds | 1 | 156 | 0 | **199** | 0 | 27 | 7 | 167 |
|  |  | 2 | 300 | 19 |  | 29 | 218 | 124 | **309** |
|  |  | 3 | 0 | 142 |  | 28 | 0 | **275** |  |
|  |  | 4 | 63 | **164** |  | 150 | 33 |  |  |
|  |  | 5 | **200** |  |  | 85 | 185 |  |  |
|  |  | 6 |  |  |  | 31 | **526** |  |  |
|  | Dioestrus with blinds | 1 | 0 | 0 | 0 | **24** | 0 | 0 | 22 |
|  |  | 2 | 0 | 0 | 42 |  | 0 | 0 | **58** |
|  |  | 3 | 0 | **15** | 0 |  | 0 | 0 |  |
|  |  | 4 | 0 |  | 0 |  | 0 | **28** |  |
|  |  | 5 | 0 |  | 0 |  | **1** |  |  |
|  |  | 6 | 0 |  | 0 |  |  |  |  |
|  | Oestrus without blinds | 1 | 30 | 14 | 22 | NA | 14 | **137** | 135 |
|  |  | 2 | 19 | 42 | 36 | NA | 15 |  | **340** |
|  |  | 3 | 66 | **91** | 46 | NA | 65 |  |  |
|  |  | 4 | 140 |  | **160** | NA | 32 |  |  |
|  |  | 5 | 93 |  |  | NA | **187** |  |  |
|  |  | 6 |  |  |  | NA |  |  |  |
|  | Dioestrus without blinds | 1 | 61 | 57 | **75** | NA | 41 | 45 | 0 |
|  |  | 2 | 91 | **135** |  | NA | 31 | 20 | 0 |
|  |  | 3 | 0 |  |  | NA | **199** | 42 | 0 |
|  |  | 4 | **59** |  |  | NA |  | 43 | 2 |
|  |  | 5 |  |  |  | NA |  | **90** | 0 |
|  |  | 6 |  |  |  | NA |  |  |  |
| 18 | Oestrus with blinds | 1 | 0 | 32 | 50 | 0 | 8 | **238** | 28 |
|  |  | 2 | 0 | 2 | **176** | 2 | 74 |  | 51 |
|  |  | 3 | 0 | 48 |  | 0 | **53** |  | 5 |
|  |  | 4 | 0 | **175** |  | 18 |  |  | 0 |
|  |  | 5 | 0 |  |  | **23** |  |  | 0 |
|  |  | 6 | 0 |  |  |  |  |  | 0 |
|  | Dioestrus with blinds | 1 | 0 | 10 | 0 | 8 | **88** | 0 | 0 |
|  |  | 2 | 0 | **193** | 0 | 0 |  | 0 | 0 |
|  |  | 3 | 0 |  | 0 | **73** |  | 0 | 0 |
|  |  | 4 | 0 |  | **2** |  |  | 0 | 0 |
|  |  | 5 | 0 |  |  |  |  | 0 | 0 |
|  |  | 6 |  |  |  |  |  |  |  |
|  | Oestrus without blinds | 1 | 69 | 0 | **134** | 0 | 0 | 56 | 102 |
|  |  | 2 | 34 | 52 |  | 17 | 3 | **107** | 59 |
|  |  | 3 | 105 | 8 |  | **163** | 21 |  | 99 |
|  |  | 4 | **194** | 16 |  |  | 25 |  | 81 |
|  |  | 5 |  | **125** |  |  | 16 |  | 121 |
|  |  | 6 |  |  |  |  | 34 |  | **159** |
|  | Dioestrus without blinds | 1 | **204** | 4 | 2 | 0 | 55 | 0 | 0 |
|  |  | 2 |  | 12 | 0 | 0 | **387** | 0 | 0 |
|  |  | 3 |  | 7 | 6 | 20 |  | **27** | 0 |
|  |  | 4 |  | **11** | 0 | 4 |  |  | 0 |
|  |  | 5 |  |  | 3 | **45** |  |  | 0 |
|  |  | 6 |  |  | **19** |  |  |  | 0 |
| 19 | Oestrus with blinds | 1 | 20 | 55 | 58 | 28 | 0 | **359** | 0 |
|  |  | 2 | 0 | 14 | 67 | 40 | 0 |  | **290** |
|  |  | 3 | 0 | 7 | **234** | 0 | 121 |  |  |
|  |  | 4 | 0 | 0 |  | 0 | **103** |  |  |
|  |  | 5 | 0 | **128** |  | 0 |  |  |  |
|  |  | 6 | 19 |  |  | **97** |  |  |  |
|  | Dioestrus with blinds | 1 | 0 | 7 | **63** | 0 | 0 | 14 | 0 |
|  |  | 2 | 0 | 3 |  | 0 | 0 | **6** | 0 |
|  |  | 3 | 0 | **9** |  | 6 | 0 |  | 3 |
|  |  | 4 | 0 |  |  | **15** | 1 |  | 0 |
|  |  | 5 | 0 |  |  |  | **12** |  | 3 |
|  |  | 6 | 0 |  |  |  |  |  | **4** |
|  | Oestrus without blinds | 1 | 26 | **250** | 4 | NA | 24 | 70 | 111 |
|  |  | 2 | 7 |  | 2 | NA | 6 | **191** | 69 |
|  |  | 3 | 43 |  | 4 | NA | 19 |  | **199** |
|  |  | 4 | **311** |  | 5 | NA | 8 |  |  |
|  |  | 5 |  |  | **30** | NA | 7 |  |  |
|  |  | 6 |  |  |  | NA |  |  |  |
|  | Dioestrus without blinds | 1 | 5 | 14 | 8 | NA | 10 | **59** | 0 |
|  |  | 2 | 34 | 21 | **49** | NA | 0 |  | 0 |
|  |  | 3 | 2 | 0 |  | NA | **15** |  | 0 |
|  |  | 4 | **12** | 0 |  | NA |  |  | 0 |
|  |  | 5 |  | **28** |  | NA |  |  | 6 |
|  |  | 6 |  |  |  | NA |  |  |  |
