# Supplementary material for: Cycle-specific female preferences for visual and non-visual cues in the horse (Equus caballus)
Source: PLoS One. 2018 Feb 21;13(2):e0191845. doi: 10.1371/journal.pone.0191845 (PMC5842875; doi:10.1371/journal.pone.0191845)
Supplement: S2 Table — (DOCX) [file pone.0191845.s002.docx]

**Table S2.** Completed and non-completed test series (*N*) per test type in 7 stallions and 19 mares, contacted stallions per test round (%), mean contact time ± SD (s), mean number of oestrous/ investigative/ affiliative and agonistic behaviours ± SD (*N*) of the mares per contacted stallion.

| **Test type** | **Completedtest series (*N*)** | **Non-completed test series (*N*)** | **Contacted stallions (%)** |  | **Contact time per contacted stallion(s)** | **Oestrous/ affiliative behaviours per contacted stallion (*N*)** | **Agonistic behaviours per contacted stallion (*N*)** |
| --- | --- | --- | --- | --- | --- | --- | --- |
| Oestrus with blinds | 17 | 2 | 66.9 |  | 64.3 (95.6) | 2.13 (4.02) | 0.24 (0.86) |
| Oestrus without blinds | 16 | 0 | 91.0 |  | 81.8 (86.2) | 3.06 (5.14) | 0.83 (1.67) |
| Dioestrus with blinds | 8 | 11 | 43.6 |  | 18.2 (53.8) | 0.03 (0.21) | 0.38 (1.38) |
| Dioestrus without blinds | 12 | 4 | 59.9 |  | 31.8 (66.9) | 0.01 (0.07) | 0.69 (1.25) |
|  |  |  |  |  |  |  |  |
